# Antitumor, Antiviral, and Anti-Inflammatory Efficacy of Essential Oils from *Atractylodes macrocephala* Koidz. Produced with Different Processing Methods

**DOI:** 10.3390/molecules24162956

**Published:** 2019-08-15

**Authors:** Sihao Gu, Ling Li, Hai Huang, Bing Wang, Tong Zhang

**Affiliations:** 1 School of Pharmacy, Experiment Center for Teaching and Learning, Shanghai University of Traditional Chinese Medicine, 1200 Cai-lun Rd, Shanghai 201203, China; 2 Experimental Teaching Center of Pharmaceutical Sciences, School of Pharmacy, Fudan University, 826 Zhang-heng Rd, Shanghai 201203, China; 3 Center for Pharmaceutics Research, Shanghai Institute of Materia Medica, Chinese Academy of Sciences, 501 Hai-ke Rd, Shanghai 201203, China

**Keywords:** *Atractylodes macrocephala* Koidz., essential oils, atractylone, processing methods, GC/MS, pharmacodynamic effect

## Abstract

*Atractylodes macrocephala* Koidz. has been used as an invigorating spleen drug for eliminating dampness and phlegm in China. According to recent researches, different processing methods may affect the drug efficacy, so we collected *A. macrocephala* from the Zhejiang Province, produced with different processing methods, crude *A. macrocephala* (CA) and bran-processed *A. macrocephala* (BA), then analyzed its essential oils (EOs) by GC/MS. The results showed 34 components representing 98.44% of the total EOs of CA were identified, and 46 components representing 98.02% of the total EOs of BA were identified. Atractylone is the main component in *A. macrocephala*. Compared with CA, BA has 46 detected compounds, 28 of which were identical, and 6 undetected compounds. Pharmacodynamic results revealed that the EOs of CA and atractylone exhibited more effective anticancer activity in HepG2, MCG803, and HCT-116 cells than the EOs of BA; while the EOs of BA exhibited simple antiviral effect on viruses H3N2, both the EOs and atractylone show anti-inflammatory activity by inhibiting the lipopolysaccharide (LPS)-induced nitric oxide (NO) production in ANA-1 cells.

## 1. Introduction

*Atractylodes macrocephala* Koidz. (*Baizhu* in Chinese), a perennial herb from the Compositae family, is a drug commonly used in traditional Chinese medicine. It has been used as a food and medicinal herb in China for thousand years [1]. In China, *A. macrocephala* is mainly distributed in Zhejiang Province which is known as one of authentic medicinal herbs “Zhebawei” [2]. Many components, such as essential oils (EOs), sesquiterpenoids, polysaccharides, amino acids, vitamins, resins, and other ingredients have been found in *A. macrocephala* up till recently [3,4]. *A. macrocephala* has many effects, such as strengthening the spleen, benefiting vital energy, eliminating dampness, hidroschesis, and soothing fetuses [5].

In China, the methods of Chinese medicine processing became more and more common after the Song Dynasty. The crude *A. macrocephala* (CA) and bran-processed *A. macrocephala* (BA) are the two drugs that are prepared in pieces which have been widely used as a tonic in China. Clinically, the CA is mainly used for spleen dampness and diuresis while after bran-processed, it can significantly relieve dryness and tonify Qi and spleen. It is because after processing, the content of EOs with sputum effect decreased, and the content of lactones which inhibited uterine contraction increased; hence the famous doctor Zhu Danxi in the Yuan Dynasty regarded BA as the miscarriage prevention medicine for the first time in the “Danxi Heart Law” [6]. The main components of *A. macrocephala* are EOs that are made up of atractylone, orange linoleum, elemene, and isoeugenol [7]. So after bran processing, what changes in the chemical composition of *A. macrocephala* have caused such changes in its pharmacological effects?

In recent years, the research on the active ingredients of *A. macrocephala* has been focused on its polysaccharides and lactones. The research on EOs of *A. macrocephala* focuses on component analysis, and rarely involved medicinal ingredients. Pharmacological studies and clinical practice have demonstrated that *A. macrocephala* possesses various bioactivities, including diarrhea, abdominal pain, and insufficiency of the stomach, intestine, liver, kidney, or insufficiency of the spleen with abundance of dampness [8,9]. *A. macrocephala* has a regulating effect on the gastrointestinal tract and is commonly used in the treatment of digestive tract diseases [9,10]. Atractylodes lactones have antitumor effects [11,12,13], but no study has been reported on whether the EOs of *A. macrocephala* has inhibitory effect on the digestive tract tumors; the previous research of the research group found that the EOs of *Cinnamomi ramulus* has antiviral and anti-inflammatory effects [14]. So, it suddenly occurred to us that since *A. macrocephala* extract has many pharmacological activities, and the EOs are one of the main active components of *A. macrocephala*, so the EOs of *A. macrocephala* might have antitumor, antiviral, and anti-inflammatory effects. 

**Figure 1:**
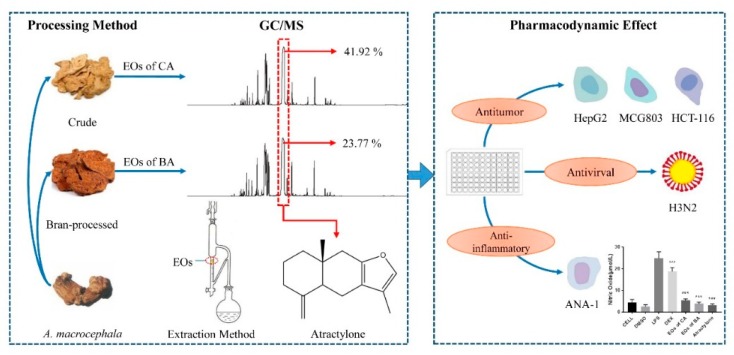
The relevance of chemical compositions in the EOs produced from *A. macrocephala* and biological activities.

Here, we collected *A. macrocephala* from the Pan’an District, Jinhua City, Zhejiang Province, China and analyzed its EOs constituents by GC/MS. In addition, for further exploitation of this plant, the possible bioactivities of the EOs of CA and BA were examined, including antitumor, antiviral, and anti-inflammatory activities, to investigate the chemical compositions and biological activities of the EOs in the effective part, and find the material basis for its function. Here, our schematic illustration of the work is revealed in Figure 1.

## 2. Results

### 2.1. Chemical Composition of Essential Oils

**Figure 2:**
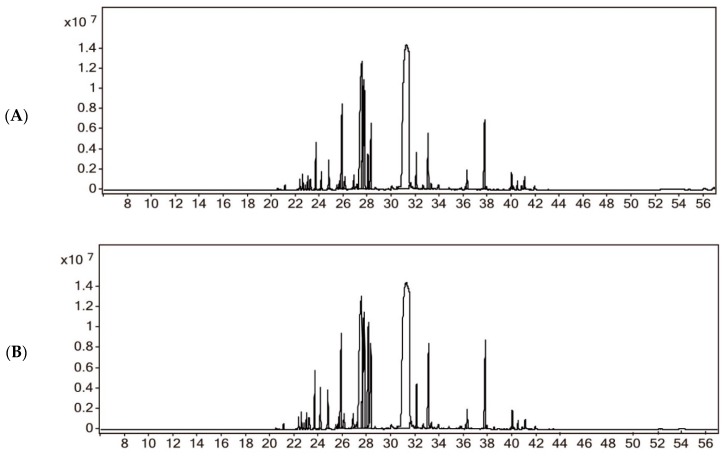
GC/MS chromatograms of the EOs of *A. macrocephala.* (**A**) GC/MS chromatograms of the EOs of crude *A. macrocephala* (CA); (**B**) GC/MS chromatograms of the EOs of bran-processed *A. macrocephala* (BA).

Hydrodistillation of CA yields 1.33% (*v*/*w*) and BA yields 1.07% (*v*/*w*) of the yellow EOs using the Chinese Pharmacopoeia appendix method (Part IV General rule 2202). We used the National Institute of Standards and Technology (NIST) database to match and select the material with the highest matching degree and reference to determine the compounds which are described in Figure 2 and Table 1. A total of 34 components representing 98.44% of the total peak areas of CA were identified. The highest content component of the EOs of CA was atractylone (41.92%). For BA, 46 components representing 98.02% of the total peak areas were identified and the highest content component of EOs of BA was atractylone (23.77%). Compared with the EOs of CA, BA had 28 same compounds (84.98% for the EOs of CA and 67.40% for the EOs of BA), 18 additional detected compounds, and 6 undetected compounds.

### 2.2. Antitumor Activity of Essential Oil from A. macrocephala

**Figure 3:**
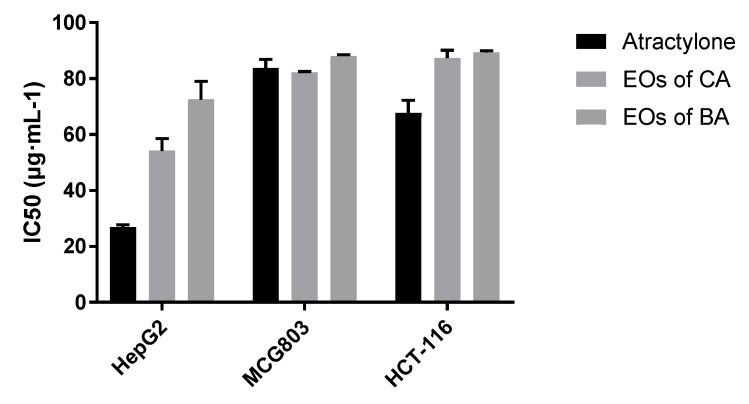
Inhibition of proliferation of HepG2, MCG803, and HCT-116 cells by atractylone, the EOs of CA and the EOs of BA (*n* = 3).

According to the results of in vitro cytotoxicity experiments, SPSS statistical software was used to treat the cell proliferation inhibition rate data, and IC_50_ values of HepG2, MCG803, and HCT-116 cells were inhibited by atractylone, the EOs of CA, and the EOs of BA. As shown in Figure 3, the atractylone inhibited the proliferation of HepG2 and HCT-116 cells with lower IC_50_ values than the EOs of CA and the EOs of BA; the EOs of CA inhibited the proliferation of HepG2 cells with lower IC_50_ values than the EOs of BA. It is speculated that atractylone has strong antitumor activity in HepG2 and HCT-116 cells, and EOs of BA show weaker antitumor effect than the EOs of CA.

### 2.3. Antiviral Activity of Essential Oil from A. macrocephala

In order to confirm the possible activity of *A. macrocephala* for antiviral activity in traditional Chinese medicine, the EOs were evaluated for its cytotoxicity and inhibition against the influenza virus H3N2 in vitro by complete cytopathic effect (CPE) inhibition rate assay, with ribavirin as the positive control. Ribavirin had good virucidal activity with TC_50_ value of 14.72 µg·mL^−1^ and no cytotoxicity at 25 µg·mL^−1^. The IC_50_ of atractylone, the EOs of CA and BA were all unmeasurable, but CPE inhibition H3N2 rates of EOs of CA were 25% at the concentration of 6.25 µg·mL^−1^, suggesting that it can partially inhibit the production of CPE of H3N2 influenza virus; the inhibition rate is <50%, suggesting that its anti-H3N2 influenza virus has limited effect. The atractylone and the EOs of BA showed no effects on the H3N2 influenza virus.

### 2.4. Anti-Inflammatory Activity of EOs from A. macrocephala

**Figure 4:**
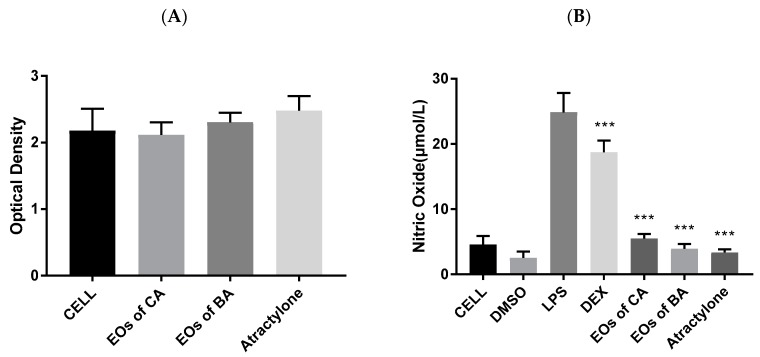
(**A**) Effects of EOs of CA, EOs of BA, and atractylone on the viability of ANA-1 cells (*n* = 3). cell: blank control; cell + EOs of CA, EOs of BA and atractylone: EOs of CA, EOs of BA and atractylone at 50 μg·mL^−1^ respectively. (**B**) NO inhibitory effects of EOs of CA, EOs of BA and atractylone on LPS-activated ANA-1 cells (*n* = 3). cell: blank control; DMSO: DMSO at the concentrations of 1:2500; LPS: LPS control; DEX: positive control of DEX at 10 μg·mL^−1^; EOs of CA, EOs of BA and atractylone: EOs of CA, EOs of BA and atractylone at 50 μg·mL^−1^ respectively; ***: *p* < 0.001 vs. LPS.

The inhibitors of NO production in macrophages via lipopolysaccharide (LPS) stimulation are considered as anti-inflammatory agents. We first investigated different concentrations of EOs of CA, EOs of BA and atractylone on the viability of ANA-1 cells by the thiazolyl blue tetrazolium bromide (MTT) method. As shown in Figure 4A, the administration of EOs of CA, EOs of BA and atractylone at 50 µg·mL^−1^ does not affect the cell activity state.

Then we investigated the NO inhibitory effects of different concentrations of LPS, dexamethasone (DEX), EOs of CA, EOs of BA and atractylone on lipopolysaccharide (LPS)-activated ANA-1 cells. As shown in Figure 4B, DMSO at the concentrations of 1:2500 has an inhibitory effect on NO secreted by cells. Compared with the normal group, the cells secreted a large amount of NO after LPS stimulation, and the content of NO secreted by the positive drug DEX decreased significantly. The DEX, EOs of CA, EOs of BA, and atractylone can significantly reduce the secretion of the NO content after stimulation. Therefore, it is believed that the EOs of CA, EOs of BA, and atractylone have anti-inflammatory activity.

## 3. Discussion

*A. macrocephala* Koidz. produced with two different processing methods, CA and BA, are commonly used in Chinese herbal medicine pieces. Previous literature studies have shown that frying with bran can reduce the dryness of the drug and improve its spleen tonifying activity [49]. EOs are plant-derived aromatic compounds that have a wide range of biological activities, but they act slowly, and they are usually unstable under light or heat, difficult to extract, and so on [50]. Based on this, we used GC/MS to study the EOs components of CA and BA. We found that the dryness nature may be related to the content of EOs, and also to the addition, no detection, or conversion of other compounds. The postoperative abortion may be related to 2-Phenylacetamide (6.32%), which has estrogenic activities [51] according to reports of *A. macrocephala* after bran-processed and does not exist in the EOs of CA.

The results of antitumor activity studies showed that the EOs of CA showed better activity in gastric cancer, intestinal cancer, and liver cancer cells. We studied the EOs components of CA and BA, and found that β-elemene and 3,7-guaiadiene are unique ingredients of the EOs of CA. β-elemene reduced cell viability and induced apoptosis in HCT116 and HT29 cells and caused cell cycle arrest at the G2 phase and induced apoptosis of SGC-7901 cells through a mitochondrial-dependent apoptotic pathway [52,53]. The EOs in Phoebe hui Cheng ex Yang exhibited significant antitumor properties, with 3,7-guaiadiene as the main constituent in the EOs [54], the content of 3,7-guaiadiene in the EOs of CA is 9.57%. So we can speculate that the antitumor activity of the EOs of CA is better than that of the EOs of BA, and the experimental results showed the same conclusion.

Anti-inflammatory experiments showed that EOs of BA has better activity. Some scholars have found that the chemical composition of EOs extracted from Brazilian propolis exhibited antibacterial activity, with γ-elemene as the main component [55]. The EOs isolated by the microwave-ultrasonic apparatus from *Teucrium pruinosum* leaves which contains agarospirol, the most abundant component (45.53%), can be a suitable candidate for use as a novel anticancer, anti-inflammatory, and antioxidant medication [56]; the content of agarospirol is 6.25% in the EOs of BA. Therefore, we believe that the anti-inflammatory ability of the EOs of BA may be stronger than that of the EOs of CA, and the experimental conclusion is reasonable.

Atractylone, the main compound in both EOs of CA and BA, can reduce allergic rhinitis (AR) clinical symptoms and biomarkers including rub scores, total IgE, histamine, prostaglandin D2, thymic stromal lymphopoietin, interleukin (IL)-1β, IL-4, IL-5, IL-6, IL-13, tumor necrosis factor-α, cyclooxygenase-2, intercellular adhesion molecule-1, and macrophage inflammatory protein-2, which suggested that atractylone is a potential therapeutic agent for AR, so atractylone has a good immune and anti-inflammatory activity [57].

NO as a free radical in the body, is associated with the occurrence of various intestinal diseases such as intestinal inflammation and increased intestinal mucosal permeability [58]. Literature research shows that reduction of tight proteins such as claudins (Cldn), Zona occludin-1 (ZO1), and occludin (Ocln) can cause inflammatory bowel disease (IBD) [58,59,60]. The experimental results of this study showed that the EOs of *A. macrocephala* and atractylone can significantly reduce the NO content secreted by LPS after ANA-1 cells stimulation, and have good anti-inflammatory activity, but little research has been done on its anti-inflammatory mechanism. It is believed that *A. macrocephala* is a spleen-requiring herb in Chinese medicine. These results revealed that the EOs of *A. macrocephala* has a good inhibitory effect on the gastrointestinal tumor cells. Based on this, we intend to design a follow-up experiment to explore the anti-inflammatory effect of the EOs on gastrointestinal inflammation and its anti-inflammatory mechanism. The preliminary results showed that atractylone reduces the intestinal inflammatory response, suggesting that its anti-inflammatory mechanism may be related to Cldn, ZO1, and Ocln in cells, and the specific anti-inflammatory mechanism needs further experimental verification.

## 4. Materials and Methods

### 4.1. Plant Materialsand Chemicals

*A. macrocephala* Koidz. were purchased from Pan’an District, Jinhua City, Zhejiang Province at the beginning of 2019, and were identified by Jun-Song Li, the senior experimenter of Teaching and Experimental Center of Shanghai University of Traditional Chinese Medicine. Preparation of crude *A. macrocephala*: After harvesting fresh medicinal herbs, wash, evenly slice, and dry at room temperature. Preparation of bran-processed *A. macrocephala*: The crude *A. macrocephala* were prepared according to the 2015 edition of the Pharmacopoeia of the People’s Republic of China. According to the ratio of crude *A. macrocephala*: sprinkle the candied bran (10:1, g/g) into a hot pot, add the crude *A. macrocephala* when it is smoked, fry until yellow brown, and escape the aroma, remove, sieve to remove the bran.

MDCK, ANA-1, and influenza viruses H3N2 was kindly provided by Teaching Center of Pharmaceutical Sciences, School of Pharmacy, Fudan University (Shanghai, China). HepG2, MCG803, and HCT-116 cells were provided by Dalian Mei-lun Biotechnology Co., Ltd. (Liaoning, China).

Atractylone (HPLC ≥ 95%) was purchased from Shanghai Xi-Jian Biological Technology Co., Ltd. (Shanghai, China); Ethyl acetate (Analytical Reagent) was purchased from Sinopharm Chemical Reagent Co., Ltd. (Shanghai, China); Fetal bovine serum (FBS), dulbecco’s modified eagle medium (DMEM), DMSO, LPS, DEX, Cell Counting Kit-8 (CCK-8) and MTT was purchased from Sigma-aldrich Chemical Reagent Co., Ltd. (Shanghai, China).

### 4.2. Extraction Methods

The crude and bran-processed *A. macrocephala* were smashed through the No. 2 sieve, weighed 100 g, placed in a 1000 mL round bottom flask, and 500 mL of water and several glass beads were added. After shaking and mixing well, they were connected with the essential oil tester and the reflux condenser, filled the water at the upper end of the condensation tube till the graduated portion of the essential oil tester and overflowed into the flask. The electric jacket was heated slowly to boiling and was kept for about 5 h until the amount of oil in the measuring device no longer increased; then stop heating, put it for a while, open the piston at the lower end of the measuring device, slowly release the water to the upper end of the oil layer up to 5 mm above the 0 line. Leave it for more than 1 h, then turn on the piston to lower the oil layer until its upper end was flush with the scale 0 line, read the volatile oil amount, and calculate the content (%, *v*/*w*) of the volatile oil in the test sample.

### 4.3. Chemical Components Analysis by GC/MS

GC/MS analysis of the test solution was performed on a gas chromatograph (HP-7890, Agilent Technologies, Santa Clara, CA, USA) coupled with a mass selective detector (HP-5977, Agilent Technologies) equipped with an HP-5MS fused silica column (30 m × 0.25 mm, 0.25 μm). The oven temperature was kept at 60 °C for 5 min, then warmed to 250 °C at 4 °C/min and held at this temperature for 5 min. A splitless injection was performed with helium gas at a flow rate of 0.9 mL/min as the carrier gas. The spectrometer uses an electronic shock (EI) mode with a scan range of 40 to 400 *m*/*z*, an ionization energy of 70 eV, and a scan rate of 0.2 s. The transmission line temperature and ion source temperature were 280 °C and 230 °C, respectively [30]. For analysis purpose, EOs of CA, EOs of BA and atractylone were dissolved in ethyl acetate.

### 4.4. Effects on Liver Cancer, Gastric Cancer, and Intestinal Cancer Cells

Logarithmic growth phase cells were seeded in 96-well culture plates at a cell number of 1 × 10^4^ cells·mL^−1^. After the cell density reached 75%, different concentrations of EOs were added and allowed to act for 24 h. Then add CCK-8 to each well, react at 37 °C for 24 h, discard the supernatant and add 100 μL of DMSO per well, shake well, and measure the optical density OD of each well at 450 nm.

### 4.5. Anti-H3N2 Virucidal Activity

#### 4.5.1. Sample Cytotoxicity Test

MDCK cells were seeded onto 96-well culture plates and cultured for 24 h. After forming cell monolayers, different concentrations of sample dilutions were added. After 72 h of incubation, cell morphology changes were observed under microscope and cell viability was determined by MTT assay.

#### 4.5.2. In vitro Antiviral Virus Test

MDCK cells were seeded onto 96-well culture plates and cultured for 24 h to form a cell monolayer. The cells were infected with influenza virus solution for 2 h, and then replaced with the maximum non-toxic concentration (concentration of cell activity ≥ 90%). After the sample was diluted for 72 h, the CPE was observed under a microscope to determine the inhibitory effect of the sample on influenza virus.

### 4.6. Anti-Inflammatory Activity

#### 4.6.1. MTT Method to Investigate the Effect of Test Drugs on Cell Viability

A single cell suspension was prepared, 1000 rpm for 5 min, and the supernatant was discarded. The cells were resuspended in 1 mL of 10% FBS medium, counted with the gentian violet [61], and the cell concentration was adjusted to 2 × 10^6^ cells·mL^−1^; evenly inoculate the cells in a 96-well cell culture plate, add 100 μL of the cell suspension to each well, and incubate for 24 h at 37 °C in a 5% CO_2_ saturated humidity incubator. After incubation, the cells were infected with different concentrations of EOs of CA, EOs of BA and atractylone (50 μg·mL^−1^). After co-culture for 24 h, add 50 μL of MTT solution at 5 mg·mL^−1^ to each well, shake well, continue to culture for 4 h, carefully aspirate the supernatant, add 200 μL of DMSO, shake well, and measure the absorbance of each well at 570 nm.

#### 4.6.2. Inhibition of ANA-1 Cell Inflammatory Model Induced by LPS Stimulation

NO production was assessed indirectly by the quantification of nitrite and nitrate according to the Griess reaction [62,63]. DEX was selected as a positive control [64]. Each tested sample was dissolved in DMSO, and diluted with fresh FBS-free DMEM media to final concentration with DMSO (1:2500). The ANA-1 macrophages were seeded in 96-well plates (2 × 10^6^ cells·mL^−1^) and co-incubated with samples EOs of CA, EOs of BA and atractylone (50 μg·mL^−1^) and LPS (0.25 μg·mL^−1^). After incubation at 37 °C for 24 h, the culture supernatant was mixed with 50 μL Griess Reagent to determine the NO production. Absorbance was measured at 570 nm using a microtiter plate reader.

## 5. Conclusions

The EOs of BA can partially inhibit the production of CPE of H3N2 influenza virus; the order of antitumor activity is atractylone, the EOs of CA, and the EOs of BA; the DEX, EOs of CA, EOs of BA and atractylone can significantly reduce the secretion of the NO content after stimulation, so it is believed that the EOs of CA, EOs of BA and atractylone have anti-inflammatory activity.

## Figures and Tables

**Table 1:** Chemical compositions and content * of the EOs of CA and BA.

No.	Retention (min)	Compounds	Molecular Formula	EOs of CA		EOs of BA		CAS No.	Reference
				Area (%)	Similarity	Area (%)	Similarity		
1	21.085	α-Guaiene	C_15_H_24_	ND		0.13	87.5	3691-12-1	[15]
2	22.327	α-Amylcinnamyl alcohol	C_14_H_20_O	0.35	87.8	0.29	87.9	101-85-9	[16]
3	22.561	Berkheyaradulene	C_15_H_24_	0.54	87.8	0.42	87.7	65372-78-3	[17]
4	22.769	Eremophylene	C_15_H_24_	ND		0.15	83.7	10219-75-7	[18]
5	22.987	α-Gurjunene	C_15_H_24_	0.48	93.1	0.97	92.8	489-40-7	[19]
6	23.189	β-Isocomene	C_15_H_24_	ND		0.26	71.1	74311-15-2	[17]
7	23.236	(−)-β-Caryophyllene	C_15_H_24_	ND		0.28	70	87-44-5	[20]
8	23.674	Isoledene	C_15_H_24_	1.85	96.3	1.75	96.3	95910-36-4	[7]
9	24.135	γ-Elemene	C_15_H_24_	0.64	93.5	1.14	93.7	29873-99-2	[21]
10	24.753	α-Caryophyllene	C_15_H_24_	1.21	92.5	1.07	92.5	6753-98-6	[22]
11	25.622	1,2,3,4,4a,7-Hexahydro-1,6-dimethyl-4-(1-methylethyl)naphthalene	C_15_H_24_	0.33	81.1	0.34	83.2	16728-99-7	[23]
12	25.850	Cedrol	C_15_H_26_O	ND		0.14	51.6	77-53-2	[24]
13	25.864	β-Selinene	C_15_H_24_	4.88	95.4	4.36	95.3	17066-67-0	[25]
14	26.085	trans-Nuciferol	C_15_H_22_O	0.69	76	0.67	78.6	39599-18-3	[26]
15	26.829	Calarene	C_15_H_24_	0.84	91.5	0.74	91.7	17334-55-3	[27]
16	27.136	Zingiberene	C_15_H_24_	0.33	76.1	0.25	65.8	495-60-3	[28]
17	27.441	(−)-Norbornenone	C_7_H_8_O	1.06	57.5	ND		16620-79-4	[29]
18	27.459	1,2,3,6-Tetramethylbicyclo[2.2.2] octa-2,5-diene	C_12_H_18_	1.68	58.5	ND		62338-43-6	[30]
19	27.486	2-Methoxy-4-methyl-1-(1-methylethyl)benzene	C_11_H_16_O	ND		0.55	60.9	1076-56-8	[30]
20	27.486	3,7-Guaiadiene	C_15_H_24_	9.57	75.7	ND		6754-04-7	[31]
21	27.497	Eudesma-4(14),11-diene	C_15_H_24_	5.34	72.6	5.38	70.2	17066-67-0	[30]
22	27.500	1-Heptanal	C_7_H_14_O	ND		1.74	57.9	111-71-7	[24]
23	27.507	1-Adamantylethanol	C_12_H_20_O	ND		3.66	61.4	6240-11-5	[32]
24	27.519	2-Phenylacetamide	C_8_H_9_NO	ND		6.32	64.3	103-81-1	[33]
25	27.674	Eudesma-3,7(11)-diene	C_15_H_24_	5.57	80.6	4.36	79.6	6813-21-4	[34]
26	27.708	Caryophyllene	C_15_H_24_	0.57	61.9	ND		87-44-5	[30]
27	27.721	β-Himachalene	C_15_H_24_	ND		0.19	61.4	1461-03-6	[30]
28	27.723	Isolongifolene	C_15_H_24_	4.33	81.3	3.04	75.9	1135-66-6	[35]
29	27.732	β-Eudesmol	C_15_H_26_O	ND		0.77	54.3	473-15-4	[36]
30	27.738	*trans*-2-Heptenal	C_7_H_12_O	ND		0.21	61.8	18829-55-5	[37]
31	27.756	(9E,12E)-9,12-Octadecadienoic acid methylester	C_19_H_34_O_2_	ND		0.41	76.8	2566-97-4	[24]
32	28.089	γ-Gurjunene	C_15_H_24_	1.31	95	4.37	94.6	22567-17-5	[38]
33	28.297	Aromadendrene	C_15_H_24_	3.23	91.1	3.29	91.9	489-39-4	[7]
34	29.978	β-Vatirenene	C_15_H_22_	0.37	78.5	0.35	77.4	27840-40-0	[7]
35	31.173	Atractylone	C_15_H_20_O	41.92	95	23.77	95	6989-21-5	[39]
36	31.239	(*Z*)-3-decen-1-ol	C_10_H_20_O	ND		8.99	78.2	10340-22-4	[30]
37	31.514	Agarospirol	C_15_H_26_O	ND		6.25	77.9	1460-73-7	[40]
38	31.559	β-Elemene	C_15_H_24_	0.25	79	ND		515-13-9	[41]
39	31.616	4,11,11-Trimethyl-8-methylenebicyclo[7.2.0] undec-4-ene	C_15_H_24_	ND		0.16	80.3	13877-93-5	[42]
40	32.066	10S,11S-Himachala-3(12),4-diene	C_15_H_24_	1.54	94.3	1.38	94.4	60909-28-6	[43]
41	32.611	Dehydroaromadendrene	C_15_H_22_	0.29	81.5	0.24	83.1	698388-95-3	[30]
42	33.039	4,5-Dehydroisolongifolene	C_15_H_22_	ND		0.45	79.5	1246777-02-5	[36]
43	33.072	Aristolone	C_15_H_22_O	2.69	87.1	3.58	82.7	6831-17-0	[44]
44	33.283	3,7,11-Trimethyl-dodeca-2,4,6,10-tetraenal	C_15_H_22_O	0.34	78.2	0.28	81.2	13832-89-8	[45]
45	33.865	Spathulenol	C_15_H_24_O	0.34	76.7	ND		6750-60-3	[7]
46	33.874	3,4,7,8-Tetrahydro-8,8,9,9-tetramethyl-2H-2,4a-methanonaphthalene	C_15_H_22_	ND		0.25	77.9	67517-14-0	[46]
47	36.250	Velleral	C_15_H_20_O_2_	1.02	85.3	0.70	84.2	50656-61-6	[47]
48	37.741	α-Curcumene	C_15_H_22_	3.65	89.2	3.95	88.8	644-30-4	[7]
49	39.958	Procerin	C_15_H_18_O_2_	0.83	72.3	0.67	74.2	552-96-5	[48]
50	40.439	8,9-dehydro-9-formyl-Cycloisolongifolene	C_16_H_22_O	0.31	69	0.25	68.8	1206188-76-2	[7]
51	41.022	3a,5,6,7,8,8a,9,9a-Octahydro-5,8a-dimethyl-3-methylenenaphtho[2-b]furan-2(3*H*)-one	C_15_H_20_O_2_	0.72	89.8	0.36	89.4	80367-94-8	[48]
52	41.861	1,2,3,3a,4,5-Hexahydro-1,1,4,4-tetramethyl-2,3b-methano-3bH-cyclopenta[1,3]cyclopropa[1,2]benzene-6-carboxaldehyde	C_16_H_22_O	0.23	68.7	0.13	67.9	59820-24-5	[48]
Total				98.44		98.02			

* ND = Not Detected.
